# A novel dinucleotide variant at 5′ splice sites in the *F8* gene causes exon 19 skipping in a Chinese family with hemophilia A

**DOI:** 10.3389/fgene.2025.1686184

**Published:** 2025-10-16

**Authors:** Xunzhao Zhou, Qi Yang, Qiuli Chen, Sheng Yi, Shengkai Wei, Jingsi Luo, Dahua Meng, Zailong Qin, Shujie Zhang

**Affiliations:** ^1^ Guangxi Key Laboratory of Birth Defects Research and Prevention, Guangxi Key Laboratory of Reproductive Health and Birth Defects Prevention, Maternal and Child Health Hospital of Guangxi Zhuang Autonomous Region, Nanning, China; ^2^ Department of Genetic and Metabolic Central Laboratory, Maternal and Child Health Hospital of Guangxi Zhuang Autonomous Region, Nanning, China; ^3^ Guangxi Clinical Research Center for Pediatric Diseases, Maternal and Child Health Hospital of Guangxi Zhuang Autonomous Region, Nanning, China; ^4^ Prenatal Diagnosis Center, Maternal and Child Health Hospital of Guangxi Zhuang Autonomous Region, Nanning, China

**Keywords:** *F8*, hemophilia A, exon 19 deletion, 5′ splice sites variant, novel variant, dinucleotide variant, minigene splicing assays

## Abstract

**Background:**

Hemophilia A is a rare, severe X-linked recessive inherited hemorrhagic disorder caused by *F8* gene dysfunction, which is characterized by spontaneous or post-traumatic bleeding tendencies. The pathogenic variants identified in the *F8* gene contribute to prenatal diagnosis and genetic counseling services for patients and their families.

**Methods:**

We used inverse shifting-PCR (IS-PCR), direct DNA sequencing, bioinformatics predictions, cDNA sequencing, and minigene splicing assays to explore candidate variants in a Chinese family with hemophilia A. The identified variant was classified in accordance with ACMG/AMP guidelines.

**Results:**

A novel c.6115+5_6115+6delinsAG variant at 5′ splice sites (5’ss) in exon 19 was identified in a 14-year-old Chinese boy with hemophilia A by DNA sequencing, which is inherited from his asymptomatic carrier mother. Multiple bioinformatics prediction tools, including SD-Score, information content (Ri), varSEAK, and RDDC RNA splicer, predicted that this variant might affect the normal pre-mRNA splicing. Both cDNA sequencing and minigene splicing assays proved that the variant led to exon 19 skipping in the *F8* gene, which was ultimately classified as pathogenic according to the ACMG/AMP guidelines.

**Conclusion:**

The c.6115+5_6115+6delinsAG variant in the *F8* gene is considered to be responsible for hemophilia A in this family. This dinucleotide variant located at 5’ss of the gene is initially reported. Our study has expanded the mutation spectrum of *F8* and provided a basis for prenatal and clinical diagnosis.

## 1 Introduction

Hemophilia A (OMIM: 306700), a rare X-linked recessive inherited hemorrhagic disorder with a prevalence rate of approximately 1 in 5,000 live male births, is characterized by the deficiency of the coagulation factor VIII (FVIII) due to a wide spectrum of pathogenic variants in the *F8* gene (Gitschier et al., 1984). Based on residual FVIII activity (FVIII: C), this disorder is clinically classified into severe (FVIII: C<1%), moderate (FVIII: C1-5%), and mild (FVIII: C6-40%) forms, accounting for approximately 60%, 10%, and 30% of all affected individuals, respectively (Bolton-Maggs and Pasi, 2003; Peyvandi et al., 2013). Affected patients typically exhibit spontaneous bleeding with significant clinical heterogeneity, including joint pain, swelling, post-traumatic or post-surgical bleeding, hemarthrosis, muscle hematomas, and so on (Bolton-Maggs and Pasi, 2003). Disease diagnosis relies on clinical manifestations, laboratory examinations, and genetic mutation analysis.

The *F8* gene located in the X-chromosome terminal q28 region spans approximately 186 kb and contains 26 exons. It encodes a precursor protein consisting of a hydrophobic signal peptide and a maturation protein with A1-A2-B-A3-C1-C2 structural domains. The mature protein found in peripheral blood is a heterodimer composed of a light chain with the A3-C1-C2 structural domains and a heavy chain with the A1-A2-B structural domains (Vehar et al., 1984). Different variant types in the *F8* gene can result in varying degrees of FVIII deficiency. So far, over 2000 variants have been recorded in disease-related databases, such as the Human Gene Mutation Database, the ClinVar Database, and the Factor VIII Gene Variant Database, including point mutations, insertions, deletions, splicing site mutations, and inversions. The inversion of intron 22 (Inv22) and intron 1 (Inv1) contributed to about half of the patients with severe hemophilia A (Castaldo et al., 2007).

In this study, we identified a novel dinucleotide variant at 5′ splice sites (5’ss) in a Chinese asymptomatic carrier female and her 14-year-old son with hemophilia A by direct DNA sequencing, bioinformatics predictions, cDNA sequencing, and minigene splicing assays, which can be responsible for hemophilia A in this family.

## 2 Materials and methods

### 2.1 Patients and clinical informations

**FIGURE 1:**
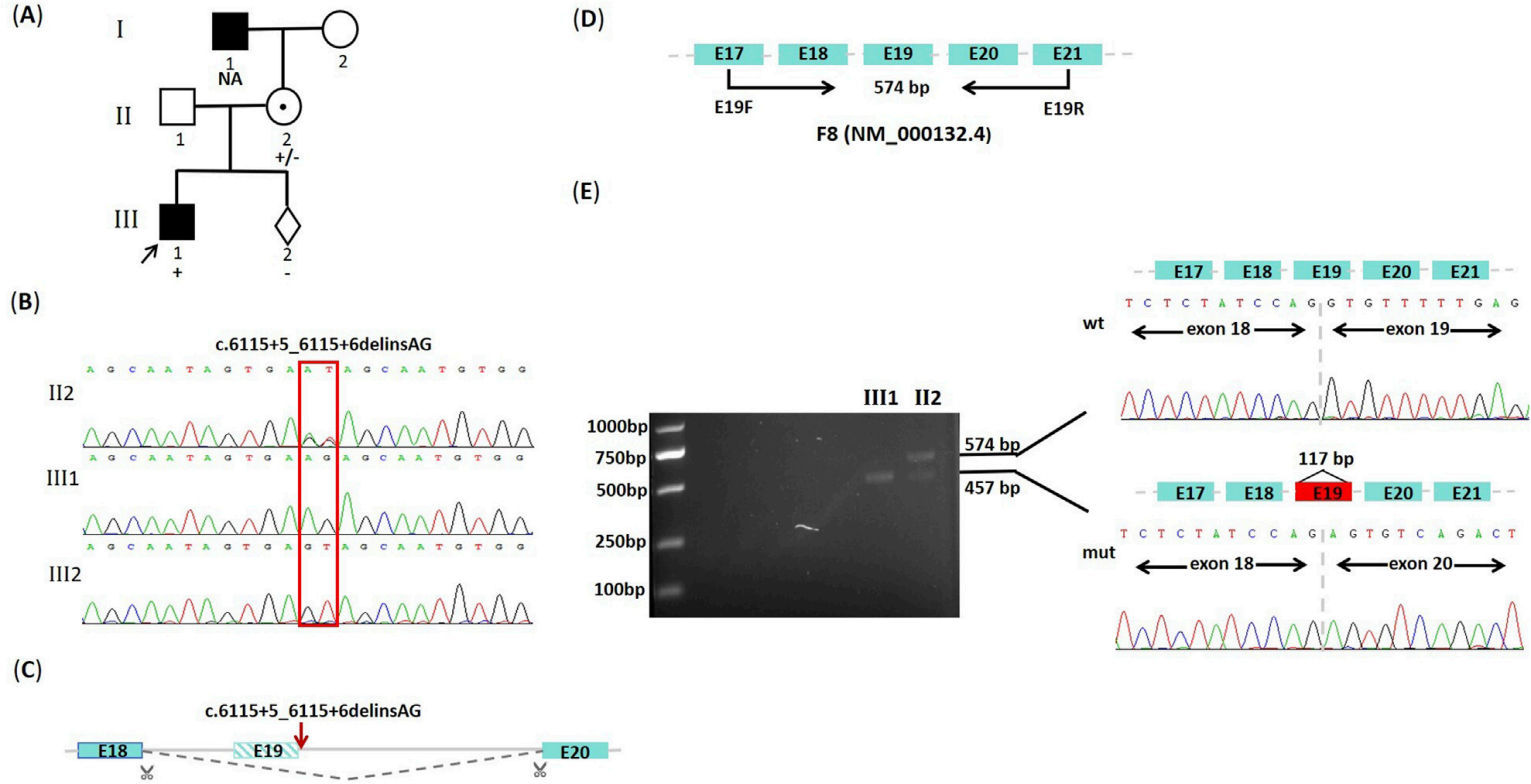
The family pedigree, predictive splicing model, and mutation analysis. **(A)** The family pedigree of hemophilia A. The proband (III1) is indicated by an arrow, and the black-filled square represents disease-affected patients (I1, III1). The NA means genotype unknown. The +/−, +, and - represent the heterozygous mutation, the hemizygous mutation, and wild genotype, respectively. **(B)** DNA sequencing results of the *F8* gene. The hemizygous **(C)**. 6115+5_6115+6delinsAG variant is identified in the proband (III1). The female (II2) is a heterozygous carrier, and the genotype of her fetus (III2) is wild. **(C)** Predictive splicing model from the RDDC RNA splicer. **(D)** Schematic diagram of cDNA sequencing for the *F8* gene (NM_000132.4) in this study. The expected size for the wild-type amplification product is 574 bp. **(E)** Agarose gel electrophoresis and Sanger sequencing results of cDNA amplification products. The electrophoresis result on the left figure shows that both the wild-type (wt) band and the shorter mutant-type (mut) band are observed in the female (II2), whereas only the mutant-type (mut) band is observed in the proband (III1). The schematic diagram of mRNA splicing and the sequencing results corresponding to the two bands are presented in the figure on the right.

The proband is a 14-year-old boy diagnosed with severe hemophilia A at a local hospital about 10 years ago based on his clinical features, coagulation abnormalities and residual FVIII: C. At present, we have not obtained original and definitive FVIII: C of the boy yet. Thereafter, he developed bilateral ear pain, arthralgia, joint swelling, hematuria, pale skin, and lower extremity pain, and received intermittent treatment with human coagulation FVIII. His mother is 33 years old and asymptomatic, who referred to the Maternal and Child Health Hospital of Guangxi Zhuang Autonomous Region for the genetic diagnosis during her pregnancy of 17 weeks. Family history investigation revealed that the maternal grandfather exhibited symptoms associated with hemophilia (Figure 1A). This study was approved by the Medical Ethics Committee of the Maternal and Child Health Hospital of Guangxi Zhuang Autonomous Region (Ethical approval number: 2022-2-16), and informed consent was obtained from the mother of the proband.

### 2.2 Genomic DNA extraction

We collected 2 mL of peripheral blood samples from the proband and his mother for inverse shifting-PCR (IS-PCR) and DNA sequencing. Once the genetic diagnosis was confirmed, 2 mL of umbilical cord blood was obtained via transabdominal cordocentesis for fetal DNA sequencing. Genomic DNA was extracted from peripheral blood and fetal cord blood leukocytes following the instruction of Lab-Aid DNA kit guidelines (Zeshan Biotechnology, Xiamen, China).

### 2.3 IS-PCR

Inv22 and Inv1 in the *F8* gene are relatively common variants for severe hemophilia A. As previously described (Yi et al., 2020; Rossetti et al., 2008), the IS-PCR containing three amplification tests (Inv22 diagnostic test, Inv22 complementary test, and Inv1 diagnostic test) was used to identify Inv22, Inv1, and intron 22 deletion (Del22) by1.5% agarose gel electrophoresis.

### 2.4 DNA sequencing

DNA sequencing for the entire coding region of the *F8* gene, including all 26 exons and adjacent intron sequences, was performed to screen for point mutations and small deletions/insertions. The PCR reaction was carried out with Taq DNA polymerase (Takara Biotechnology, Dalian, China) in a total volume of 25ul, including 9.5ul H_2_O, 12.5ul r-Taq Master Mix, 1ul forward primer, 1ul reverse primer, and 1ul genomic DNA. The Primer sequences are listed in Supplementary Table S1. The PCR program was composed of the following steps: denaturation at 95 °C for 30 s, annealing at appropriate temperature for 30 s, and extension at 72 °C for 60 s, repeated for a total of 35 thermal cycles. All amplification products were subsequently subjected to Sanger sequencing in an ABI 3500 genetic analyzer. The ClinVar database (https://www.ncbi.nlm.nih.gov), the Human Gene Mutation Database (https://www.hgmd.cf.ac.uk/ac/), and the Factor VIII Gene Variant Database (https://www.factorviii-db.org/) were utilized to assess the clinical significance of variants. Ultimately, the variant was classified in accordance with the ACMG/AMP guidelines.

### 2.5 *In-silico* analysis

The SD-Score online website (https://www.med.nagoya-u.ac.jp/neurogenetics/SD_Score/sd_score.html) provides prediction results for SD-Score and information contents (R_i_). The SD-Score was utilized to predict the potential effects of 5′splice site (5′ss) mutations by using a common logarithm calculated from the frequency of a specific 5′ss in the human genome. This tool accepts both wild-type and mutant-type 5′ss sequences spanning nine nucleotides from exon −3 to intron +6 positions and returns differences in the SD-Score (ΔSD-Score) with a sensitivity of 97.1% and specificity of 94.7%, respectively (Sahashi et al., 2007; Ohno et al., 2018). Subsequently, the potential impact on authentic splice sites was further assessed using the varSEAK online site (https://varseak.bio/). The visualized splicing prediction software, Rare Disease Data Center (RDDC) RNA splicer (https://rddc.tsinghua-gd.org/), was used to further explore the possible splicing patterns. This AI model tool predicts alternative splicing sites within mRNA sequences by learning canonical splicing patterns (Jaganathan et al., 2019; Zhang et al., 2023). Three-dimensional models of the protein were established using the PyMol software to observe structural changes induced by the mutation and to predict potential factors contributing to these alterations.

### 2.6 cDNA sequencing analysis

To identify the effect on pre-mRNA splicing, peripheral blood samples were collected from the proband and his mother for cDNA sequencing analysis. Firstly, the RNA was extracted from leucocytes by the standard Trizol method (Life Technology, MA, United States). Total RNA was then reverse-transcribed into cDNA in accordance with the First-strand cDNA Synthesis Kit (Takara Biotechnology, Dalian, China), followed by amplification for exon 19 with the following primers: Forward, 5′-CAT​GGG​AGA​CAA​GTG​ACA​GT-3′; Reverse, 5′-GAT​CCG​GAA​TAA​TGA​AGT​CTG-3′. The PCR reaction was conducted with TransTaq^®^ HiFi DNA Polymerase (TransGen Biotech, Beijing, China) in a total volume of 25 ul, including 16.7 ul H_2_O, 0.3 ul HiFi DNA Polymerase, 2.5 ul HIFI Buffer II, 2.0 ul dNTPs (2.5 mM), 1 ul forward primer, 1 ul reverse primer, and 1.5 ul cDNA template. The PCR amplification was performed according to the following procedure: denaturation at 95 °C for 30 s, annealing at 60 °C for 50 s, and extension at 72 °C for 60 s, repeated for a total of 35 thermal cycles. Finally, PCR products were separated on a 1.5% agarose gel and validated by Sanger sequencing.

### 2.7 Minigene recombinant vector construction

To further validate effects of the variant on pre-mRNA splicing, *in vitro* minigene splicing assays were performed. We constructed two types of minigene recombinant vectors including pcMINI and pcMINI-C. Firstly, nested PCR was performed by using two pairs of primers (119065-F and 121868-R, 119429-F and 121448-R) with normal genomic DNA as the template. Then, pcMINI wild-type fragment (1406bp) and pcMINI-C wild-type fragment (1417bp) containing two enzymatic sites (KpnI and XhoI) were obtained after PCR amplification with primers pcMINI-*F8*-KpnI-F and pcMINI-*F8*-XhoI-R, pcMINI-C-*F8*-KpnI-F and pcMINI-C-*F8*-XhoI-R, respectively. As previously reported (Liu et al., 2025), overlap extension PCR (OE-PCR) was used to obtain mutant-type fragments containing the mutation site (c.6115+5_6115+6delinsAG) and two enzymatic sites by using *F8*-mut-F and *F8*-mut-R as mutation primers. Primer information is listed in Supplementary Table S2. The above amplification products were sequentially subjected to gel electrophoresis, gel recovery, and purification to obtain purified target DNA fragments. The wild-type fragments, mutant-type fragments, and plasmids were digested at 37 °C for 45 min using both KpnI and XhoI restriction endonucleases. The digested products were then purified and ligated using DNA ligase at 22 °C for 90 min. Finally, the ligation products were transformed into DH5αfollowed by incubation overnight at 37 °C. Several monoclonal colonies were randomly selected for PCR and Sanger sequencing and the identified colonies were subjected to plasmid DNA extraction using the Rapid Plasmid Mini Kit (Simgen Biotechnology, Hangzhou, China).

### 2.8 Minigene transcriptional analysis

The four constructed recombinant plasmids were transiently transfected into HeLa and HEK-293T cell lines by liposome transfection. After 48 h of transient transfection, total RNA was extracted from each of the 8 cell samples by Trizol (TaKaRa Biotechnology, Dalian, China), respectively. ABScript III RT Master Mix for qPCR with gDNA Remover (ABclonal Biotechnology, Wuhan, China) was used for reverse transcription into cDNA according to the kit instructions. The PCR amplification was performed using the minigene flanking primers (pcMINI-F and pcMINI-R, pcMINI-C-F and pcMINI-C-R). Finally, the RT-PCR products were separated by agarose gel electrophoresis and each band of products was collected separately for Sanger sequencing.

## 3 Results

### 3.1 IS-PCR and genomic DNA sequencing

Considering that the proband was a patient with severe hemophilia A, we initially performed the IS-PCR study. However, the gel electrophoresis of IS-PCR showed negative results in both the proband and his mother (Supplementary Figure S1). After excluding the presence of Inv22, Inv1, and Del22, we used direct DNA sequencing to find candidate disease-causing variants. Except for a dinucleotide variant at 5’ss in exon 19 (c.6115+5_6115+6delinsAG), there were no other non-benign variants identified in exon regions and classical splice sites (+1, +2). Sequencing results confirmed that the variant was hemizygous in the proband and inherited from his mother (Figure 1B). This variant was absent from both the dbSNP database and the gnomAD population database. Furthermore, it was not documented in the ClinVar database, the Human Gene Mutation Database, or the Factor VIII Gene Variant Database. The dinucleotide variant at 5’ss of the *F8* gene was reported for the first time, which was originally classified as a variant of uncertain significance (PM2_Supporting, PS1_Moderate, and PP3) by ACMG/AMG guidelines.

### 3.2 *In-silico* analysis

According to the predictions from the SD score online website, the SD score and Ri score for the mutant-type were calculated as −5.879 and 2.355, respectively. The difference in SD score (ΔSD Score) and Ri (ΔRi) between the wild-type and the mutant-type were −2.447 and −4.412, respectively, which suggested that a potentially aberrant splicing event might occur (Table 1). The predictions from varSEAK showed that the variant resulted in a significant decrease in score for the authentic splice site (c.6115+1G), which could lead to loss of normal splicing function and exon skipping (Table 1). The RDDC RNA splicer predicted that the following splicing pattern might occur: the entire exon 19 was removed resulting in a deletion of 117bp (Figure 1C).

**TABLE 1:** *In-silico* predictions for the c.6115+5_6115+6delinsAG variant.

SD-score prediction								
Wt.Seq			Mut.Seq			Difference^a^		
Seq.^b^	SD score	Ri	Seq.^b^	SD score	Ri	ΔSD Score	ΔRi	Prediction^c^
ATAgtgagt	−3.432	6.767	ATAgtgaag	−5.879	2.355	−2.447	−4.412	Aberrant
VarSEAK splice site prediction								
Predicted positions		Class	Score	ΔScore	MaxEntScan		ΔMaxEntScan	
c.6115+1		5	+22.56%	−80.87%	6.97		−14.62	
c.6115+5_6115+6delinsAG			−58.31%		−7.65			

^a^ The differences in the SD-Score (ΔSD-Score) and Ri (ΔR) are calculated by subtracting the wild-type (wt) score from the mutant-type(mut) score, respectively.

^b^ Sequence analysis accepts nine nucleotides from −3, to +6 positions at 5’ss. Mutation sites are marked by underlining.

^c^ The prediction results are classified as aberrant or normal. The aberrant result is determined according to the following rules: 1) ΔSD-Score < −0.34 and SD-Score < −2.9; 2) ΔSD-Score < −0.34, SD-Score > −2.9, and ΔRi > −1.45, otherwise the result is judged as normal.

### 3.3 cDNA sequencing analysis

To further confirm whether this variant adversely affected pre-mRNA splicing, we performed cDNA sequencing for exon 19 based on bioinformatics predictions. The expected size for the wild-type PCR product is 574bp (Figure 1D). The agarose gel electrophoresis of RT-PCR products showed two different bands in this female: a wild-type band with 574bp and a shorter abnormal band (mutant-type band), but only the mutant-type band, instead of the wild-type band, was observed in the proband. Sequencing results confirmed that the shorter band resulted from an aberrant transcript with the entire removal of exon 19 (117bp), which indicated that the c.6115+5_6115+6delinsAG variant affected the pre-mRNA splicing and led to exon 19 skipping *in vivo* (Figure 1E). This variant was ultimately re-classified as pathogenic (PVS1(RNA), PM2_Supporting, and PS1_Moderate) by ACMG/AMG guidelines.

### 3.4 Minigene transcription analysis

**FIGURE 2:**
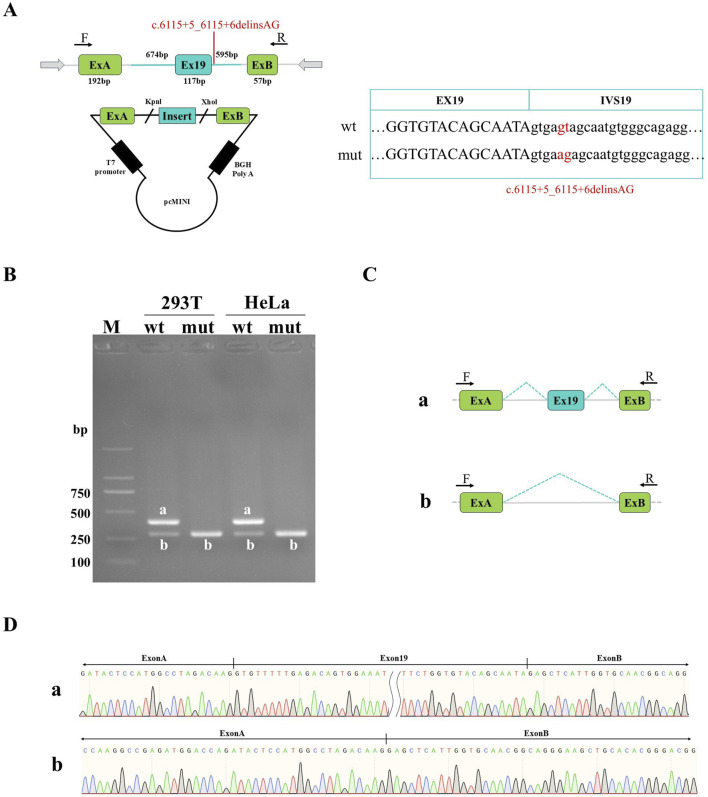
Detection results of pcMINI recombinant vectors. **(A)** Minigene construction strategy diagram. The strategy involves inserting partial intron18 (674 bp)-Exon19 (117 bp)-partial intron19 (595 bp) into pcMINI vectors. **(B)** Agarose gel electrophoresis of RT-PCR products in HeLa and HEK-293T cells. The band a is the wild-type band, and the band b is the aberrant splicing band with exon19 skipping. **(C)** Splicing schematic of minigene analysis; **(D)** Sanger sequencing results of corresponding bands.

**FIGURE 3:**
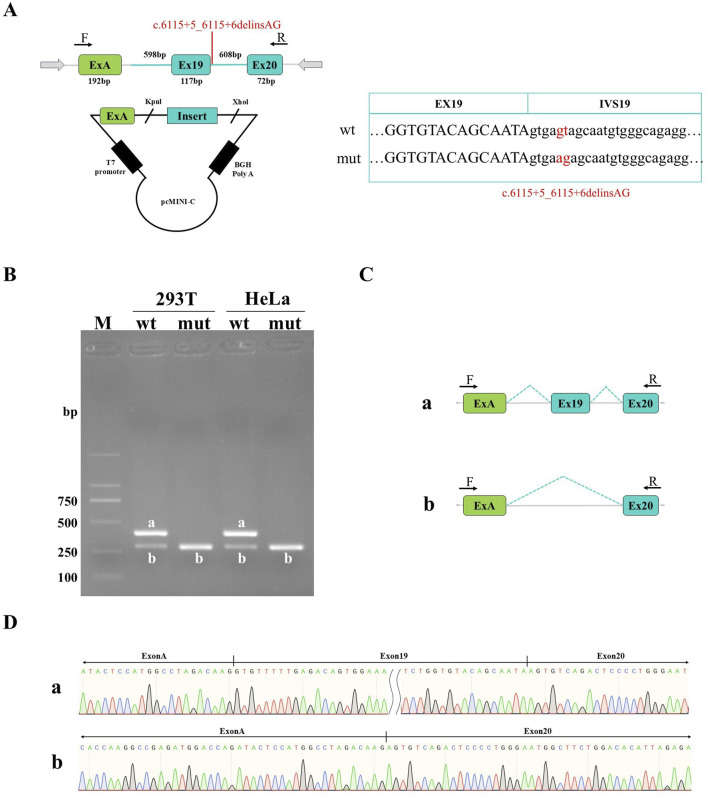
Detection results of pcMINI-C recombinant vectors. **(A)** Minigene construction strategy diagram. The strategy involves inserting partial intron18 (598 bp)-Exon19 (117 bp)-intron19 (608 bp)-Exon20 (72 bp) into pcMINI-C vectors. **(B)** Agarose gel electrophoresis of RT-PCR products in HeLa and HEK-293T cells: The band a is the wild-type band, and the band b is the aberrant splicing band with exon19 skipping. **(C)** Splicing schematic of minigene. **(D)** Sanger sequencing results of corresponding bands.

Sanger sequencing results of both pcMINI and pcMINI-C recombinant plasmids showed that wild-type and mutant-type fragments were inserted into the corresponding vectors, indicating that the minigene recombinant vectors were successfully constructed (Figures 2A, 3A). Agarose electrophoresis of RT-PCR products from the wild-type revealed two bands in HeLa and HEK-293T cells: the bright band a with the expected size and the small grey band b, whereas electrophoresis for the mutant-type only showed the small band b (Figures 2B, 3B). Sanger sequencing analysis showed that the band a is a normal splicing band, while the band b is an abnormal splicing band with the exon 19 skipping (Figures 2C,D, 3C,D). This variant was identified as c.5999_6115del and p.Gly2000_Lys2039delinsGlu at the cDNA and protein level. The exon19 skipping in the *F8* gene resulted in an in-frame deletion of 39aa in the protein sequence, which might produce a truncated protein with a length of 2312aa. The results were consistent in two vectors as well as in 2 cell lines. Our study has confirmed that c.6115+5_6115+6delinsAG disturbed the normal splicing of pre-mRNA in cultured cells *in vitro*, which was consistent with our findings *in vivo*.

## 4 Discussion

While the proband was diagnosed with hemophilia A at the local hospital, further genetic testing was not initially performed at that time. Unfortunately, we still lacked the result of initial level of plasma FVIII: C tested in the local hospital, which was critical for disease diagnosis and severity classification. Following a summary analysis of relevant literature and disease databases, we found a total of 19 patients with c.6115+1_ 6115+6 variants in intron 19. All affected individuals suffered from severe hemophilia A, with available FVIII: C levels <1% (Table 2). Our study suggested the c.6115+5_6115+6delinsAG variant found in the proband was responsible for hemophilia A. The female came to our hospital for excluding the possibility of carrier for her fetus. Based on c.6115+5_6115+6delinsAG variant, we performed a prenatal genetic diagnosis for the fetus, and DNA sequencing analysis showed that the fetus did not carry the same variant in cord blood DNA. Ultimately, the female gave birth to a asymptomatic male infant, which further confirmed the validity of our genetic diagnosis.

**TABLE 2:** Summary of patients with c.6115+1_ 6115+6 variants in intron 19 of the *F8* gene.

Variant	Amino-acid change	Number of patients	FVIII: C(%)	Severity	References
c.6115+1G>A	Exon19 skipping^a^	1	<1	Severe	Lombardi et al. (2021)
c.6115+1_6115+3del	Exon19 skipping^b^	1	<1	Severe	Factor VIII Gene Variant Database
c.6115+2T>C	Exon19 skipping^a^	2	<1	Severe	Lombardi et al., 2021; ClinVar database
c.6115+2T>G	Exon19 skipping^b^	1	<1	Severe	Factor VIII Gene Variant Database
c.6115+2T>A	Exon19 skipping^b^	1	<1	Severe	Factor VIII Gene Variant Database
c.6115+3G>T	Exon19 skipping^a^	3	<1	Severe	Lombardi et al. (2021)
c.6115+3G>C	Exon19 skipping^b^	1	<1	Severe	Factor VIII Gene Variant Database
c.6115+3_6115+6del	Exon19 skipping^b^	1	<1	Severe	Factor VIII Gene Variant Database
c.6115+4A>G	Exon19 skipping^a^	1	NR	Severe	Lombardi et al. (2021)
c.6115+4A>C	Exon19 skipping^b^	1	<1	Severe	Factor VIII Gene Variant Database
c.6115+4delA	Exon19 skipping^b^	1	<1	Severe	Factor VIII Gene Variant Database
c.6115+5G>A	Exon19 skipping^a^	2	<1	Severe	Lombardi et al., 2021; Tavassoli et al., 1997
c.6115+5G>C	Exon19 skipping^b^	1	<1	Severe	Factor VIII Gene Variant Database
c.6115+5_6115+6delinsAG	Exon19 skipping^a^	1	NR	Severe	This study
c.6115+6T>A	Exon19 skipping^a^	1	<1	Severe	Lombardi et al. (2021)

^a^ The amino acid change has been functionally demonstrated to be exon 19 skipping.

^b^ The amino acid change is predicted to result in exon 19 skipping by the RDDC RNA, splicer.

NR, not reported.

Pre-mRNA splicing primarily involves constitutive splicing and alternative splicing, which is a fundamental process in the regulation of gene expression. Alternative splicing regulates gene expression at the post-transcriptional level through selecting different splicing patterns, generating a variety of mature mRNA splice isoforms that translate multiple proteins with distinct structures and functions (Liu et al., 2022). Variants at any position within a gene have the potential to induce aberrant splicing in the following major ways: activation of cryptic splice sites, creation of new splice sites, and disruption of normal splicing recognition. The consequences of aberrant splicing are most likely to be entire exon skipping, loss of an exon fragment, and inclusion of an intron fragment, especially when mutations occur within consensus splice site sequences including 5′ss, 3′splice sites (3′ss), branch point, polypyrimidine tract sequences, splicing silencers, and splicing enhancers (Baralle and Baralle, 2005). The appearance of splicing variants destroy normal mRNA sequences and proteins function, which can lead to human disease. However, the mechanisms associated with pre-mRNA splicing remain to be further explored (Baralle and Baralle, 2005; Nikom and Zheng, 2023; Rogalska et al., 2023).

In general, variants at 5′ss are most likely to cause single exon skipping and activation of cryptic splicing sites, especially the former (Buratti et al., 2007). The determinants of these two splicing patterns may be multifaceted, including affected exon length, splice sites strength, RNA secondary structure, open reading frame conservation, DNA/RNA sequence near the splice sites, the abundance of potential cryptic 5′ss, the cis-acting elements, and so on (Fox-Walsh et al., 2005; Krawczak et al., 2007; Carranza et al., 2022). However, splicing outcomes in different genes are not always consistent and maybe even contradictory. A study on splicing defects in the *NF1* gene suggests that the splicing outcome resulting from 5′ss variants is predominantly associated with the presence of cis-acting elements in pre-mRNA (Wimmer et al., 2007). The existence of high-density and strong cryptic 5′ss within the exon in conjunction with a strong authentic 3’ss may serve as the primary determinant for activating cryptic 5′ss instead of exons skipping. However, another study shows that the presence of cryptic splice sites may not affect splicing outcomes caused by intron +1 G-A mutation in the *DMD* gene (Habara et al., 2009). The author hypothesizes that both the strength of the acceptor splice site and the affected exon length are determinative.

Some variants have been reported to be associated with aberrant splicing events in exon 19, but these splicing mechanisms are not always the same (Tavassoli et al., 1997; Wang et al., 2020; Lombardi et al., 2021). The c.5999-27A>G variant in intron 18 leading to exon 19 skipping is identified in two unrelated patients with moderate hemophilia A. The SVM-BPfinder, a branch point prediction tool, suggests the variant may disrupt the potential branch point in intron 18 and affect the splice site recognition in exon 19 (Wang et al., 2020). Several variants at 5′ss including intron +1 to +6 positions disrupt the donor splice site and have been confirmed to result in exon 19 skipping. By increasing the ability to bind hnRNP F/H, some missense variants, such as p.Arg2016Gly, p.Arg2016Leu, and p.Glu2018Gly, can lead to exon 19 skipping as well (Lombardi et al., 2021). This suggests that a few missense variants have the dual effect on both protein function and splicing process, which may explain why they result in more severe phenotypes. Interestingly, the exon 19 is one of those poorly defined exons that may contain some supposed splicing regulatory elements that promote exon inclusion. In brief, aberrant splicing events in exon 19 appear to cause the same splicing result, that is the entire exon skipping, rather than the activation of cryptic splicing sites or the creation of new splice sites.

Although many single-nucleotide variants are reported to cause splicing defects, the effect of dinucleotide variants at 5′ss on the splicing process in the *F8* gene has not been investigated. The possibility that there are some compensatory mechanisms attenuating abnormal splicing cannot be excluded. The consensus sequence in the U2-dependent 5’ss includes (C/A) AG|GT (A/G) AGT representing the −3 to +6 positions in the exon-intron boundary, where the dinucleotide GT is highly conserved (Zhang, 1998). Pre-mRNA splicing in eukaryotes involves two main processes: the U1 snRNA recognizes and binds the 5’ss, and the U6 snRNA pairs with nucleotides at positions +2, +5, and +6 (Wan et al., 2020; Wilkinson et al., 2020; Martelly et al., 2021). Contiguous base pairing contributes to the correct recognition by U1 snRNA and U6 snRNA, whereas the 5′ss sequence is not always completely complementary to them. When this occurs, other complementary bases at the 5′ss may make a major contribution to correct splicing selection, which may be related to the base-pairing compensation mechanism. The “two-point” analysis indicates that during U1 snRNA recognition, noncomplementary nucleotides (NCp-nucs) at positions −3, −2, and −1 are compensated by complementary nucleotides (Cp-nucs) at positions +4, +5, and +6, whereas NCp-nucs at positions +4, +5 and +6 are compensated by Cp-nucs at positions −3, −2, −1 and +3 (Sahashi et al., 2007). In this article, the wild-type 5’ss sequence is ATA|GTGAGT, where the NCp-nucs at positions −3, −2, −1 can be compensated by Cp-nucs at intron positions +4, +5 and +6. The mutated 5’ss sequence is ATA|GTGAAG, which is mispaired with bases at positions −3, −2, −1, +3, +5, +6 to U1 snRNA and +5, +6 to U6 snRNA. Compared with the wild-type sequence, the NCp-nucs at exonic positions −3, −2, and −1 in the mutated sequence are not compensated by the Cp-nucs at intronic positions +5, and +6. Although exon recognition depends on multiple factors, the inability of U1 snRNA and U6 snRNA to correctly recognize and bind the mutated 5’ss likely disrupts normal splicing of exon 19, which is one of the main factors leading to exon skipping.

**FIGURE 4:**
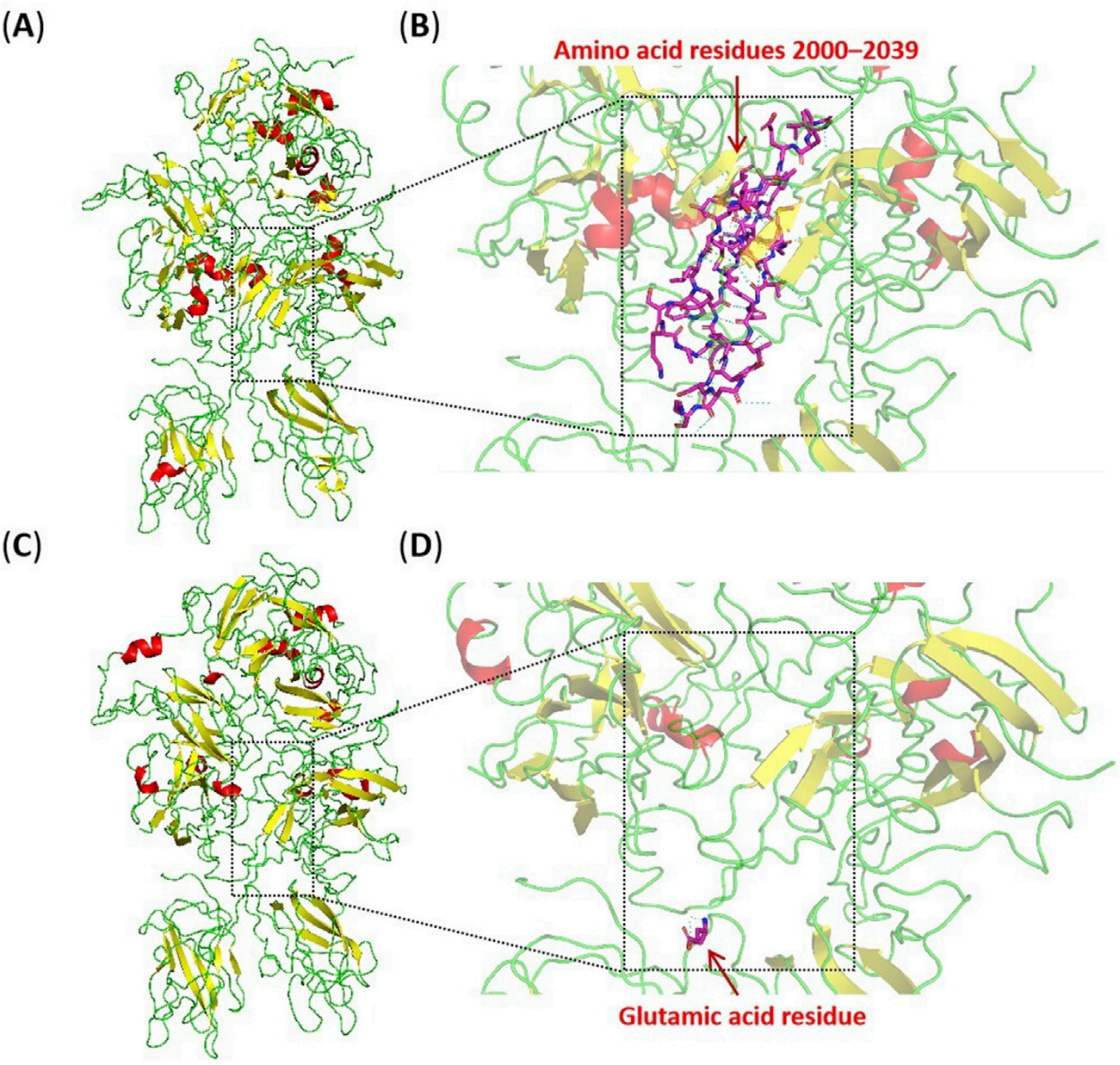
Three-dimensional models of the wild-type and truncated protein visualised by the PyMol software based on PDB 2R7E. **(A)** The integral structural model of the wild-type protein. **(B)** The partial structural model of the wild-type protein, with purple stick-shaped amino acids representing residues 2000–2039 in the wild-type protein. **(C)** The integral structural model of the truncated protein. **(D)** The partial structural model of the truncated protein, with the purple stick-shaped amino acid representing the inserted glutamic acid residue in the truncated protein. The region within the square dotted line in the figure indicates the location of the residues 2000–2039. The red, yellow, and green structures represent alpha-helixes, beta-sheets, and loops, respectively.

The abnormal splicing event produces a new transcript without 117 bases of exon 19 rather than the presence of NMD (nonsense-mediated mRNA decay). This transcript may be translated into a truncated protein where the corresponding 39 amino acids in the A3 domain are removed (Lombardi et al., 2021). The A3 domain of coagulation FVIII where some pathogenic missense variants have been reported is mainly involved in binding von Willebrand factors and forming a stable compound to escape degradation in plasma (Summers et al., 2011; Bloem et al., 2013; Yi et al., 2020). The truncated protein lacks part of the A3 domain (residues 2000–2039) and is inserted with a glutamic acid residue, which will result in significant disruption of the protein three-dimensional structure. The A3 and C1 domain can form extensive and closely interacting regions, involving multiple aromatic, aliphatic, and hydrophilic residues, which effectively maintain the structural stability of domains. Using the PyMol software to visualise the three-dimensional structure of the protein, it is observed that the truncated region involves a beta-sheets, an alpha-helix, and several loops. Hydrogen bonds and hydrophobic interactions within relevant regions are disrupted, impairing the correct folding and structural stability of the protein (Figure 4). It is speculated that this may lead to protein misfolding and loss of stability, thereby affecting the interaction between the *F8* protein and other molecules (including von Willebrand factors and FIXa), and hindering the formation of stable complexes. The truncated protein destroys normal blood clotting function, which may lead to disease.

## 5 Conclusion

In conclusion, we have identified a novel splicing variant c.6115+5_6115+6delinsAG in the Chinese boy with hemophilia A and his asymptomatic mother, which may be the pathogenicity of hemophilia A in this family. This dinucleotide variant at 5’ss of the *F8* gene is initially reported. Our research has expanded the mutation spectrum of the *F8* gene and provided a basis for prenatal and clinical diagnosis as well.

## Data Availability

The original contributions presented in the study are publicly available in the Mendeley Data repository. This data can be found here: https://data.mendeley.com/datasets/tfdh737bh8/1.

## References

[B1] Baralle D. Baralle M. (2005). Splicing in action: assessing disease causing sequence changes. J. Med. Genet. 42, 737–748. 10.1136/jmg.2004.029538 16199547 PMC1735933

[B2] Bloem E. Meems H. van den Biggelaar M. Mertens K. Meijer A. B. (2013). A3 domain region 1803-1818 contributes to the stability of activated factor VIII and includes a binding site for activated factor IX. J. Biol. Chem. 288, 26105–26111. 10.1074/jbc.M113.500884 23884417 PMC3764813

[B3] Bolton-Maggs P. H. B. Pasi K. J. (2003). Haemophilias A and B. Lancet 361, 1801–1809. 10.1016/S0140-6736(03)13405-8 12781551

[B4] Buratti E. Chivers M. Královicová J. Romano M. Baralle M. Krainer A. R. (2007). Aberrant 5’ splice sites in human disease genes: mutation pattern, nucleotide structure and comparison of computational tools that predict their utilization. Nucleic Acids Res. 35, 4250–4263. 10.1093/nar/gkm402 17576681 PMC1934990

[B5] Carranza F. Shenasa H. Hertel K. J. (2022). Splice site proximity influences alternative exon definition. RNA Biol. 19, 829–840. 10.1080/15476286.2022.2089478 35723015 PMC9225289

[B6] Castaldo G. D’Argenio V. Nardiello P. Zarrilli F. Sanna V. Rocino A. (2007). Haemophilia A: molecular insights. Clin. Chem. Lab. Med. 45, 450–461. 10.1515/CCLM.2007.093 17439320

[B7] Fox-Walsh K. L. Dou Y. Lam B. J. Hung S.-P. Baldi P. F. Hertel K. J. (2005). The architecture of pre-mRNAs affects mechanisms of splice-site pairing. Proc. Natl. Acad. Sci. U. S. A. 102, 16176–16181. 10.1073/pnas.0508489102 16260721 PMC1283478

[B8] Gitschier J. Wood W. I. Goralka T. M. Wion K. L. Chen E. Y. Eaton D. H. (1984). Characterization of the human factor VIII gene. Nature 312, 326–330. 10.1038/312326a0 6438525

[B9] Habara Y. Takeshima Y. Awano H. Okizuka Y. Zhang Z. Saiki K. (2009). *In vitro* splicing analysis showed that availability of a cryptic splice site is not a determinant for alternative splicing patterns caused by +1G-A mutations in introns of the dystrophin gene. J. Med. Genet. 46, 542–547. 10.1136/jmg.2008.061259 19001018

[B10] Jaganathan K. Kyriazopoulou Panagiotopoulou S. McRae J. F. Darbandi S. F. Knowles D. Li Y. I. (2019). Predicting splicing from primary sequence with deep learning. Cell 176, 535–548. 10.1016/j.cell.2018.12.015 30661751

[B11] Krawczak M. Thomas N. S. T. Hundrieser B. Mort M. Wittig M. Hampe J. (2007). Single base-pair substitutions in exon-intron junctions of human genes: nature, distribution, and consequences for mRNA splicing. Hum. Mutat. 28, 150–158. 10.1002/humu.20400 17001642

[B12] Liu Q. Fang L. Wu C. (2022). Alternative splicing and isoforms: from mechanisms to diseases. Genes (Basel) 13, 401. 10.3390/genes13030401 35327956 PMC8951537

[B13] Liu C. Zhang Y. Zhao Y. Luo H. (2025). A novel loss-of-function SYCP2 variant causes asthenoteratozoospermia in infertile males. Front. Genet. 16, 1595720. 10.3389/fgene.2025.1595720 40432880 PMC12106479

[B14] Lombardi S. Leo G. Merlin S. Follenzi A. McVey J. H. Maestri I. (2021). Dissection of pleiotropic effects of variants in and adjacent to F8 exon 19 and rescue of mRNA splicing and protein function. Am. J. Hum. Genet. 108, 1512–1525. 10.1016/j.ajhg.2021.06.012 34242570 PMC8387460

[B15] Martelly W. Fellows B. Kang P. Vashisht A. Wohlschlegel J. A. Sharma S. (2021). Synergistic roles for human U1 snRNA stem-loops in pre-mRNA splicing. RNA Biol. 18, 2576–2593. 10.1080/15476286.2021.1932360 34105434 PMC8632089

[B16] Nikom D. Zheng S. (2023). Alternative splicing in neurodegenerative disease and the promise of RNA therapies. Nat. Rev. Neurosci. 24, 457–473. 10.1038/s41583-023-00717-6 37336982

[B17] Ohno K. Takeda J.-I. Masuda A. (2018). Rules and tools to predict the splicing effects of exonic and intronic mutations. Wiley Interdiscip. Rev. RNA 9, e1451. 10.1002/wrna.1451 28949076

[B18] Peyvandi F. Kunicki T. Lillicrap D. (2013). Genetic sequence analysis of inherited bleeding diseases. Blood 122, 3423–3431. 10.1182/blood-2013-05-505511 24124085 PMC4260973

[B19] Rogalska M. E. Vivori C. Valcárcel J. (2023). Regulation of pre-mRNA splicing: roles in physiology and disease, and therapeutic prospects. Nat. Rev. Genet. 24, 251–269. 10.1038/s41576-022-00556-8 36526860

[B20] Rossetti L. C. Radic C. P. Larripa I. B. De Brasi C. D. (2008). Developing a new generation of tests for genotyping hemophilia-causative rearrangements involving int22h and int1h hotspots in the factor VIII gene. J. Thromb. Haemost. 6, 830–836. 10.1111/j.1538-7836.2008.02926.x 18284600

[B21] Sahashi K. Masuda A. Matsuura T. Shinmi J. Zhang Z. Takeshima Y. (2007). *In vitro* and *in silico* analysis reveals an efficient algorithm to predict the splicing consequences of mutations at the 5’ splice sites. Nucleic Acids Res. 35, 5995–6003. 10.1093/nar/gkm647 17726045 PMC2094079

[B22] Summers R. J. Meeks S. L. Healey J. F. Brown H. C. Parker E. T. Kempton C. L. (2011). Factor VIII A3 domain substitution N1922S results in hemophilia A due to domain-specific misfolding and hyposecretion of functional protein. Blood 117, 3190–3198. 10.1182/blood-2010-09-307074 21217077 PMC3062317

[B23] Tavassoli K. Eigel A. Pollmann H. Horst J. (1997). Mutational analysis of ectopic factor VIII transcripts from hemophilia A patients: identification of cryptic splice site, exon skipping and novel point mutations. Hum. Genet. 100, 508–511. 10.1007/s004390050543 9341862

[B24] Vehar G. A. Keyt B. Eaton D. Rodriguez H. O’Brien D. P. Rotblat F. (1984). Structure of human factor VIII. Nature 312, 337–342. 10.1038/312337a0 6438527

[B25] Wan R. Bai R. Zhan X. Shi Y. (2020). How is precursor messenger RNA spliced by the spliceosome? Annu. Rev. Biochem. 89, 333–358. 10.1146/annurev-biochem-013118-111024 31815536

[B26] Wang X. Hu Q. Tang N. Lu Y. Deng J. (2020). Deep intronic F8 c.5999-27A>G variant causes exon 19 skipping and leads to moderate hemophilia A. Blood Coagul. Fibrinolysis 31, 476–480. 10.1097/MBC.0000000000000950 32833809

[B27] Wilkinson M. E. Charenton C. Nagai K. (2020). RNA splicing by the spliceosome. Annu. Rev. Biochem. 89, 359–388. 10.1146/annurev-biochem-091719-064225 31794245

[B28] Wimmer K. Roca X. Beiglböck H. Callens T. Etzler J. Rao A. R. (2007). Extensive *in silico* analysis of NF1 splicing defects uncovers determinants for splicing outcome upon 5’ splice-site disruption. Hum. Mutat. 28, 599–612. 10.1002/humu.20493 17311297

[B29] Yi S. Zuo Y. Yu Q. Yang Q. Li M. Lan Y. (2020). A novel splicing mutation in F8 causes various aberrant transcripts in a hemophilia A patient and identifies a new transcript from healthy individuals. Blood Coagul. Fibrinolysis 31, 506–510. 10.1097/MBC.0000000000000952 32852327

[B30] Zhang M. Q. (1998). Statistical features of human exons and their flanking regions. Hum. Mol. Genet. 7, 919–932. 10.1093/hmg/7.5.919 9536098

[B31] Zhang Y. Bi S. Dai L. Zhao Y. Liu Y. Shi Z. (2023). Clinical report and genetic analysis of a Chinese neonate with craniofacial microsomia caused by a splicing variant of the splicing factor 3b subunit 2 gene. Mol. Genet. Genomic Med. 11, e2268. 10.1002/mgg3.2268 37555391 PMC10724505

