# Supplementary material for: A novel dinucleotide variant at 5′ splice sites in the F8 gene causes exon 19 skipping in a Chinese family with hemophilia A
Source: Front Genet. 2025 Oct 16;16:1686184. doi: 10.3389/fgene.2025.1686184 (PMC12571557; doi:10.3389/fgene.2025.1686184)
Supplement: Supplementary file 1 [file DataSheet1.docx]

Supplementary Material

**Table S1**. PCR primers for DNA sequencing analysis

| Exon | Sequence (5′-3′) | | Product size (bp) | Annealing temperature (°C) |
| --- | --- | --- | --- | --- |
|  | Forword | Reverse |  |  |
| 1 | AATCCTATCGGTTACTGCTTA | AGCATCACAACCATCCTAAC | 431 | 60 |
| 2 | TGGAAGCATTACTTCCAGCT | AACTGCAACCTCAAGATTGG | 277 | 60 |
| 3 | GCATGCTTCTCCACTGTGAC | ACGCCACCATTACAAAGCAC | 301 | 60 |
| 4 | TGCAGAAAGTCCGTTTCTTATG | CAGGTGAAGGAACACAAATGC | 420 | 60 |
| 5 | CTTACTGTCAAGTAACTGATG | CTTCATTCCTGAACAGTAATG | 280 | 58 |
| 6 | TCCCACTTATTGTCATGGAC | TACAGAACTCTGGTGCTGAA | 422 | 60 |
| 7 | CTTGGCAAGAGCTGTTGGTT | AATGTCCCCTTCAGCAACAC | 500 | 60 |
| 8 | ATATAGCAAGACACTCTGACATTG | AGAGAGTACCAATAGTCAAA | 336 | 58 |
| 9 | TCACTCCTTGCCTTGATTGAA | CCATTGGAGACAAGGCTGAA | 372 | 60 |
| 10 | CTAGCCTCAAATTACTATAATG | ACTTTAGACTGGAGCTTGAG | 347 | 58 |
| 11 | TGCGACTTTAGCTTCCACTT | ACTGACCTATATTGCAAACCA | 446 | 60 |
| 12 | TGCCATCGCTTTCATCATAG | CATTCATTATCTGGACATCAC | 320 | 60 |
| 13 | TCATGACAATCACAATCCAAAATAC | ATAAATGACAGCATGTGAGCTAGTG | 376 | 60 |
| 14a | CTGGGAATGGGAGAGAACCT | GTCTATTGCTCCAGGTGATGG | 505 | 58 |
| 14b | CTACTCCACATGGGCTATCCTTATC | ATAACCTACCACTCTCTGTTGACGA | 525 | 60 |
| 14c | CCTCTGAGCTTGAGTGAAGAAAATA | CCAGAGTTCAGAGAGTTCTTTCC | 560 | 60 |
| 14d | ACCAGATGCACAAAATCCAGATAT | TGAGCTGTGTGTTTCTTTGTTCTAT | 543 | 60 |
| 14e | GATACATACAGTGACTGGCACTAAG | GCAATCTGATAAGGGAGACTGAG | 481 | 62 |
| 14f | ACATGAAACATTTGACCCCGA | CGTAGGGAATAGGTCCTTCTGAT | 497 | 58 |
| 14g | GCCACAAATTCAGTCACATACAAG | GACTTCTATTTCGGGCTTATTTTGT | 477 | 62 |
| 14h | CAGATACCAAAAGAAGAGTGGAAAT | CTGGTAAAGTCAAATGTCACAAGAG | 499 | 60 |
| 15 | AGATGAAGTGGTTAACTATGC | GTGGGAATACATTATAGTCAG | 349 | 60 |
| 16 | AGCATCCATCTTCTGTACCA | TCAGTAGATTCCAGAATGACA | 526 | 54 |
| 17 | TGTCATTCTGGAATCTACTGA | CACTCCCACAGATATACTCT | 492 | 60 |
| 18 | TGTGGGAGTGGAATCCTCAT | AGCATGGAGCTTGTCTGCTT | 397 | 60 |
| 19 | TTCGCATAAACCAATGTATCTCA | GCAACCATTCCAGAAAGGAA | 272 | 60 |
| 20 | GACGTTCTCCCATTTTCATTG | GGATTCATTATCTGAGATTCTCCACCAG | 244 | 60 |
| 21 | TGTCTAGGACTAACCCAGCTGAA | TTTGAGCTTGCAAGAGGAATAAG | 216 | 62 |
| 22 | TCAGGAGGTAGCACATACAT | GTCCAATATCTGAAATCTGC | 287 | 60 |
| 23 | GTCTTATGTAGATGTTGGATG | AGTCTCAGGATAACTAGAACA | 350 | 60 |
| 24 | GCTCAGTATAACTGAGGCTG | CCCATAACCAAACTTCCTTGACAC | 352 | 60 |
| 25 | AGTGCTGTGGTATGGTTAAG | TTGCTCTGAAAATTTGGTCATA | 372 | 60 |
| 26 | GGACTACTGGAAACAACTAGAAGTG | GGATTTAGCACAAAGGTAGAAGG | 353 | 60 |

**Table S2**. PCR primers for minigene analysis

| Name | Sequence（5′-3′） |
| --- | --- |
| 119065-F | aatagatttggccaggtgcg |
| 119429-F | aagccctgtaacttttctgct |
| F8-mut-F | GTACAGCAATAgtgaAGagcaatgtgggcaga |
| F8-mut-R | tctgcccacattgctCTtcacTATTGCTGTAC |
| 121448-R | aagtatagcttcctgctgcac |
| 121868-R | actctcacgcctccttttca |
| pcMINI-F8-KpnI-F | ggtaGGTACCggataggttaaaggtaaggg |
| pcMINI-F8-XhoI-R | tttcCTCGAGggtggaacacagtaactaga |
| pcMINI-C-F8-KpnI-F | ggtaGGTACCactgtttgtgcctggcaata |
| pcMINI-C-F8-XhoI-R | TAGACTCGAGTCCATATTGTCCTGAAGCTGTA |
| pcMINI-C-F | ACTTAAGCTTatgagtgggctttggggtggccggtt |
| pcMINI-C-R | TAGACTCGAGTCCATATTGTCCTGAAGCTGTA |
| pcMINI-F | ACTTAAGCTTatgagtgggctttggggtggccggtt |
| pcMINI-R | TAGAAGGCACAGTCGAGG |


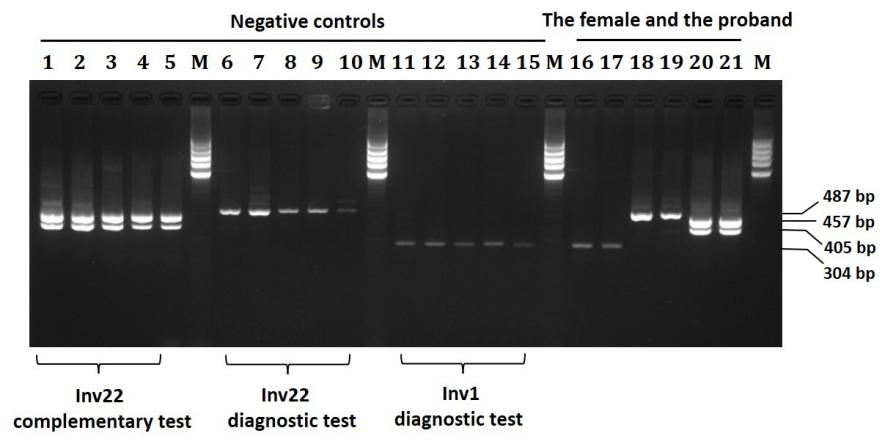


**Figure S1**. Gel electrophoresis results for IS-PCR. Lanes 1 to 5, lanes 6 to 10, and lanes 11 to 15 represent the results of the intron 22 inversion (Inv22) complementary test, Inv22 diagnostic test, and intron 1 inversion (Inv1) diagnostic test from negative controls, respectively. Lanes 16, 18, and 20 represent the results of Inv1 diagnostic test, Inv22 diagnostic test, and Inv22 complementary test from the female, respectively. Lanes 17, 19, and 21 represent the results of Inv1 diagnostic test, Inv22 diagnostic test, and Inv22 complementary test from the proband, respectively.
